# Topology assisted self-organization of colloidal nanoparticles: application to 2D large-scale nanomastering

**DOI:** 10.3762/bjnano.5.132

**Published:** 2014-08-04

**Authors:** Hind Kadiri, Serguei Kostcheev, Daniel Turover, Rafael Salas-Montiel, Komla Nomenyo, Anisha Gokarna, Gilles Lerondel

**Affiliations:** 1 Laboratoire de Nanotechnologie et d’Instrumentation Optique, Institut Charles Delaunay, CNRS UMR 6279, Université de Technologie de Troyes, 12 rue Marie Curie, BP 2060, 10010 Troyes, Cedex, France; 2 SILSEF SAS, 382 Rue Louis Rustin, Archamps Technopole, 74160 Archamps, France

**Keywords:** assisted self-organization, dislocations, patterning, polystyrene beads, single crystal

## Abstract

Our aim was to elaborate a novel method for fully controllable large-scale nanopatterning. We investigated the influence of the surface topology, i.e., a pre-pattern of hydrogen silsesquioxane (HSQ) posts, on the self-organization of polystyrene beads (PS) dispersed over a large surface. Depending on the post size and spacing, long-range ordering of self-organized polystyrene beads is observed wherein guide posts were used leading to single crystal structure. Topology assisted self-organization has proved to be one of the solutions to obtain large-scale ordering. Besides post size and spacing, the colloidal concentration and the nature of solvent were found to have a significant effect on the self-organization of the PS beads. Scanning electron microscope and associated Fourier transform analysis were used to characterize the morphology of the ordered surfaces. Finally, the production of silicon molds is demonstrated by using the beads as a template for dry etching.

## Introduction

The development of nanoscience and nanotechnology has led to a continuous miniaturization of microelectronic components. 193 nm optical-mask lithography is the driving force behind this evolution [1]. Techniques such as double patterning lithography [2], extreme UV (EUV) or electron beam have been developed or are under development to meet the needs of this miniaturization [3]. Blocking points remain to be solved for these technological solutions. In particular, photoresists show intrinsic limitations in terms of resolution, sensitivity and roughness. In addition, their industrial applications are proving to be increasingly expensive. It is therefore essential to investigate innovative, cost-effective technological approaches in order to meet the future needs of the nanotechnology industry. Taking into consideration the different problems posed by some of the above mentioned techniques, a number of other methods (e.g., the auto-organization technique) have been demonstrated for the 2D and 3D auto-organization of micro-particles on solid substrates [4–10]. The formation of self-assembled monolayers of polystyrene beads on a silicon surface has been achieved by this technique [11–12]. It is a simple, inexpensive and quick preparation method for fabrication of structures even on a large surface. However, the spontaneous process leads to the formation of micro-domain arrays, polycrystalline in nature, consisting of randomly oriented ordered regions, or grains, which limit the potential applications [13]. This issue has led to the development of a host of techniques that attempt to control the ordering of thin–film polystyrene lattice [14–17], that is, to template the position of the micro domains. In this study, we have developed a novel method of fabricating large scale ordered structures. This method combines top-down and bottom-up approaches [18]. In the top-down approach a guide post template is fabricated by an electron beam lithography (EBL) technique on a silicon surface. Thereafter, on these guide posts, self-organization of polystyrene beads is conducted (“bottom up” approach). Many articles have reported the grapho-epitaxy of self-assembled block copolymers on two-dimensional periodically patterned templates with an aim to control the orientation of the structures [19–20]. The network obtained by the self-organization of copolymer is polycrystalline and contains defects that limit its application. The purpose of using patterned substrates is to control these defects and obtain a single crystal structure on a large scale, however, this technique (grapho-epitaxy) works well only on a small scale.

In this study, we concentrated on topology-assisted self-organization by using polystyrene beads (PS) dropped on a patterned silicon substrate. We optimized the self-organization of the PS beads on the silicon substrate, wherein we observed that the deposited particles (PS beads) are very sensitive to different physico-chemical parameters. Colloidal concentration and the nature of the solvent were the two important parameters which affected the self-organization of the beads. The optimisation of these parameters allows us to produce large-scale colloidal 2D monocrystals. Another problem addressed in this study was the presence of dislocations in these PS bead patterns. By using patterned silicon substrates, we were able to minimize the presence of these dislocations resulting in the formation of single crystal structures of PS beads on the entire pre-patterned area, which is scalable depending on the technique used for pre-patterning.

## Results and Discussion

### Convective self-organization on a patterned substrate

**Figure 1:**
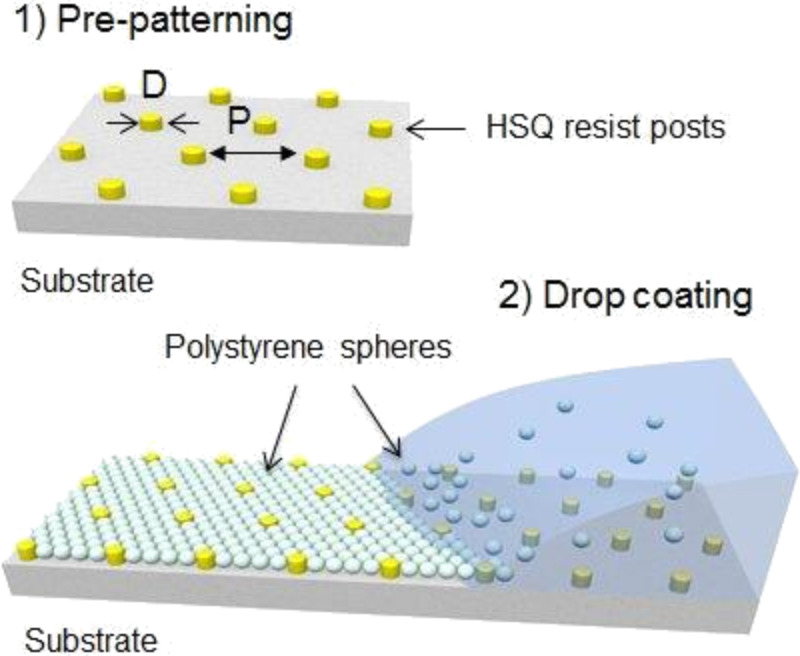
Large-scale topology assisted organization: Schematic representation of the particle (PS beads) assembly process driven by the liquid flow on a pre-patterned substrate with D and P corresponding to the post diameter and pitch, respectively.

Figure 1 illustrates the dynamics of the formation of a hexagonal array of PS beads on 2D patterned substrates. The ordering of the particles starts when the height of the water droplet containing the polystyrene beads becomes equal to the height of the particles. Direct observation shows that the main factor responsible for the ordering to occur in two dimensions is the capillary force, which is related to the evaporation rate. Therefore, the control of the evaporation rate can result in the formation of either a monolayer or multilayers [21]. The concentration (1 wt %) is chosen in order to ensure the formation of a dense mono-layered structure after the evaporation of water. A few minutes after the beginning of the experiment (droplet deposition) it was observed that the concentration of particles on the substrate increased with time, due to particle sedimentation and water evaporation. The particles come closer and form a hexagonal lattice. Previous studies have shown that these deposited particles (PS beads) are very sensitive to different physico-chemical parameters such as the nature of the solvent, the concentration of the solution, temperature, the nature of substrate, and the rate of evaporation [22]. The control of these parameters plays an essential role in the formation of single crystal structured nano (sub-micro) objects covering a large area.

**Figure 2:**
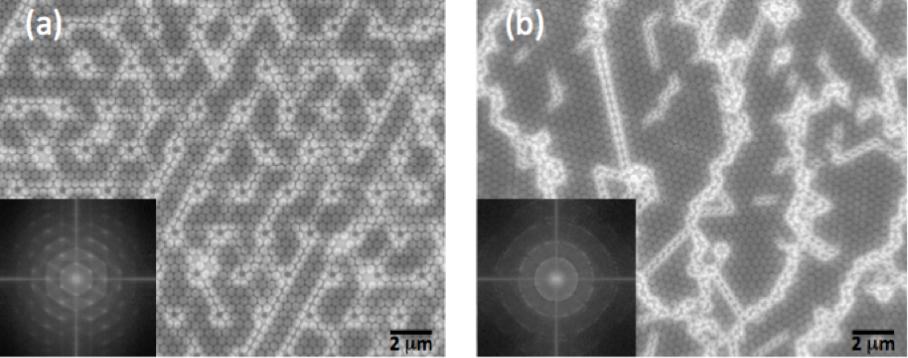
SEM images showing a comparison of (a) structures with a template (topology assisted self-organization) and (b) structures fabricated in the absence of a template (simple self-organization).

In our experiments, only the colloidal concentration and the nature of the solvent were addressed. First, we used only water as a solvent, but the drop did not cover the entire surface due to hydrophobicity. Hence, we decided to replace water by ethanol thereby increasing the wettability of the surface. With only ethanol, we were able to cover the entire surface, but the overall structure was not ordered even within a short distance. Therefore, we used a solution of 50% water and 50% ethanol. The use of this solvent mixture resulted in well-dispersed and ordered structures with the presence of micron-sized dislocations. We further decreased the concentration of ethanol to 20%. With this lesser amount of ethanol, we were able to decrease the width of the dislocations observed with the mixture of water and ethanol in a 1:1 ratio. We also changed the initial concentration of the solution which was 0.2 to 3 wt %. It was found that when the concentration is very high, the surface occupied by a multilayered structure becomes larger. If, on the other hand, the concentration is low we notice the presence of large empty spaces between the structured areas. Moreover, in the presence of a low concentration many particles adhere to the surface of the substrate before reaching the ordered regions and form small aggregates consisting of several particles. With the concentration used (1 wt %) after optimization we had a maximum thickness of 1.2 microns, which corresponds to 3 layers of beads. Moreover, multilayered assembly of the particles occurs at the edge of the sample. However, by forming patterns at the center of the sample prevents the formation of multilayers. The development of self-organization of PS beads on silicon substrates allows us to obtain a large-scale ordering (of the order of a few centimeters), but the problem of the presence of dislocations in the structure still remains. In this work, as already mentioned, our goal was to use the pre-patterned substrates as a means to form single crystal 2D structures especially on a large scale. The SEM images in Figure 2 present the typical results obtained from the assembly process. We can clearly see the strong interaction between the PS beads and the hexagonal HSQ lattice posts. PS beads will assemble in a compact hexagonal form. This structural arrangement of the beads is strongly influenced by the initial hexagonal lattice arrangement of the posts. The patterned substrate can change the shape of the meniscus of the water droplet containing PS beads and the contact angle between the droplet and the substrate, thus influencing the auto-organization process.

### Comparison of structures without template (simple self-organization) and structures with template (topology assisted self-organization)

Although self-organization presents a simple structure obtained with different grain orientations, the presence of dislocations limits its applications. So the role of assisted self-organization is to obtain a structure with a single orientation based on a hexagonal lattice of HSQ posts. When we compare the structures obtained by these two techniques, it is clear that assisted self-organization occurs in the presence of the posts, and single crystal structures of the PS beads on the entire pre-patterned area is obtained. Indeed, the presence of these posts allows us to control the self-organization and get a 2D structure with a well-defined orientation and long-range order as seen in the SEM images in Figure 2a. The spontaneous process (simple self-organization) as captured by the SEM images in Figure 2b leads to the formation of a polycrystalline structure composed of randomly oriented ordered regions. This difference in orientations is governed by the liquid flow. In the case of the patterned substrate, the flux of particles became slower because the wettability of the surface increases with the presence of the patterning. As a consequence, there is a good dispersion of the beads on the patterned substrate, resulting in a regular hexagonal arrangement. The orientation of the bead network is the same as the lattice post. As a consequence, most of the surface area is covered by a monolayer of PS beads exhibiting the same orientation. The Fourier transform confirmed that the PS bead arrays were regular, hexagonal in shape and had long-range ordering. The optimization of the structure was conducted by tuning three parameters, namely the diameter, spacing (pitch) and height of the posts. It is noteworthy that as we started with an HSQ resin of height 400 nm, the beads got deposited on top of the posts. A height of 600 nm was found to prevent this kind of deposition.

#### Effect of the distance between posts

**Figure 3:**
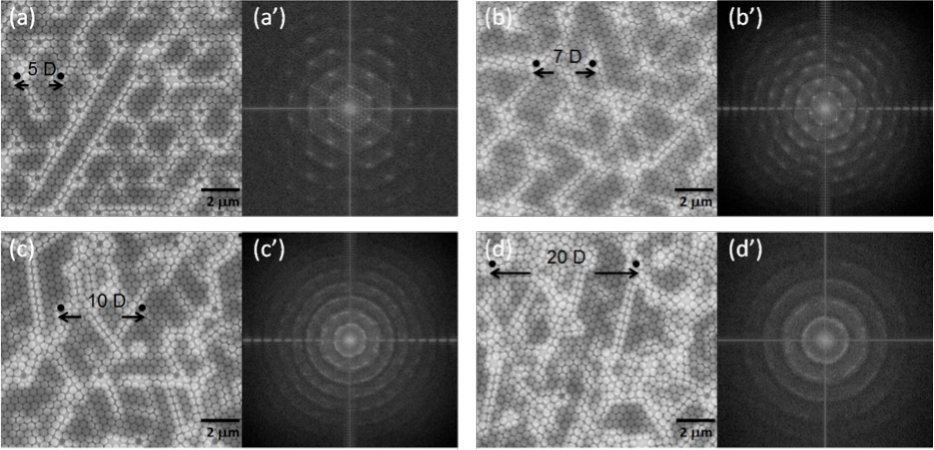
Influence of the pre-pattern pitch (post to post distance) with a fixed post diameter *D* = 390 nm. SEM images with P = (a) 5, (b) 7, (c) 10 and (d) 20 D, respectively. (a)’, (b’), (c’) and (d’) correspond to the 2D Fourier transforms of (a), (b), (c) and (d) images, respectively.

Figure 3a–d shows plane view SEM images of all the structures fabricated with a variation in the pitch (5 to 20 pitches). Figure 3a and Figure 3b are the SEM images of ordered PS spheres formed within a sparse 2D lattice of HSQ posts. Figure 3a’ and Figure 3b’ show 2D Fourier transforms representing a well-ordered, hexagonal arrangement of the PS beads between posts. The best results were obtained with 5 and 7 pitches (distance between the posts). The structures are well-oriented in these two cases. Bright lines could be caused by either fluctuation in the pre-patterned post position or the size dispersion of the beads. For 7 pitches, we observed a slight offset in the orientation of the arrangement of beads compared to the arrangement in the case of 5 pitches, but the order of the structure is maintained. In samples fabricated with pitches higher than 7, i.e., for 10 pitches and 20 pitches, a disorder in the arrangement of the PS beads is observed as seen in Figure 3c and Figure 3d. The boundaries between the different grain orientations are clearly recognizable. This disorder increases with an increasing distance between the pitches, and there is no single lattice orientation but a polycrystalline structure composed of randomly oriented regions. This result is similar to the case of simple self-organized beads structuration. Figure 3c’ and Figure 3d’ represent 2D Fourier transform of the domain positions which show the absence of long-range ordering.

#### Influence of the post diameter on the structural arrangement

**Figure 4:**
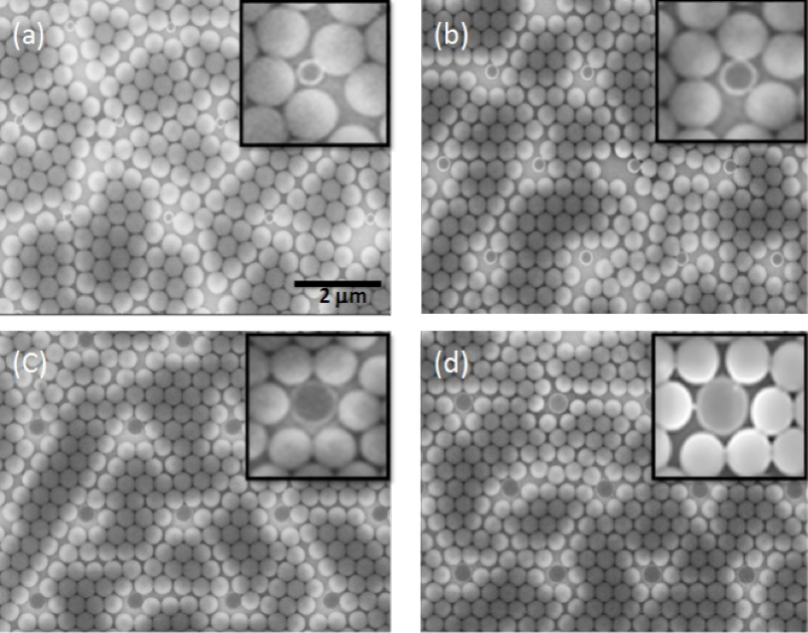
Influence of the pre-pattern post diameter: SEM images of self-assembled polystyrene beads on the post lattice template fabricated with a post diameter of (a) 195, (b) 296, (c) 390 and (d) 420 nm, respectively.

By varying the post diameter, we tried to understand its influence on the self-organization. In order to form a single crystal structure without defects, we used different post sizes: 195 nm, 296 nm, 390 nm and 420 nm, respectively. Figure 4 shows the SEM images of self-assembled PS beads on the post lattice, with post diameters equal to (a) 195 nm, (b) 296 nm, (c) 390 nm, and (d) 420 nm, respectively. The size of the post lattice clearly influences the order of the structures. When the diameter of the posts is nearly equal to the diameter of the beads, i.e., 390 nm and 420 nm, the formation of a single crystal without defects is observed. The inset in Figure 4c and Figure 4d show SEM images which exhibit a good hexagonal-shaped arrangement between the polystyrene beads and the HSQ posts. A post with diameter smaller than the bead diameter, i.e., 296 nm, leads to the formation of a polycrystalline structure with defects located near the post as clearly observed in the inset in Figure 4b. In this case, we do not observe a hexagonal arrangement of the beads surrounding the post. With a very small post diameter equivalent to 195 nm, the presence of the dislocation increases, as seen in Figure 4a. These posts appear as surface defects. They increase the dislocation density in the structure.

#### Silicon nanostructures produced by RIE etching

**Figure 5:**
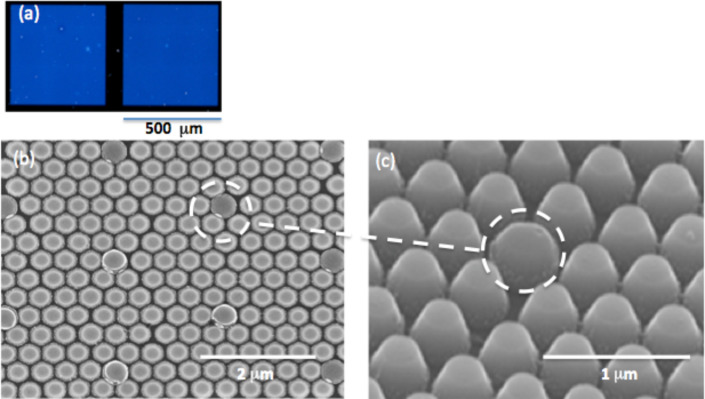
Nanomastering after a direct transfer by using RIE etching: (a) optical view (dark field) of the pre-patterned area (500 × 500 µm^2^), (b) and (c) master top and 45° tilted SEM views, respectively. Pre-patterned post leads to slightly higher structures as highlighted by the dashed circles.

The effect of dry etching on the patterned silicon has also been studied. Silicon substrates with PS beads on them are directly etched [23–24]. Figure 5 shows the SEM images of the patterned Si after RIE etching. The height of the patterns depends on the etching time. Here, the etching time was adjusted in order to have the same height for the surface either covered by the beads or the HSQ posts. A reduction of the diameter of the PS beads from 400 nm to 366 nm was observed for an etching time of 4 min.

## Conclusion

In summary, we demonstrated a simple, inexpensive and rapid procedure to produce large-scale colloidal 2D monocrystals. The technique is based on the topology assisted self-assembly of polystyrene beads (PS) on patterned silicon surfaces. The development of self-organization of PS beads on silicon substrates allows us to obtain a single crystal, which is 100 times larger compared to the ones on non-patterned Si surfaces. The actual size of the monocrystal is limited by the pre-patterned area. To yield well-defined structures, we optimized the parameters of the patterned posts. The best result was obtained for the sample with a post diameter identical to the beads diameter and a pitch corresponding to 5 to 7 times the latter. Finally, the 2D monolayers were used as etching masks to realize silicon mold. As a perspective, it is worth mentioning that this study of assisted self-organization of PS beads can be extended to a variety of substrates of different materials. This paves the way to large-scale patterning applications as an example of biosensitive surfaces useful for detecting proteins and cells. Patterned substrates using assisted self-organization can also be employed as templates for the selective growth of semiconducting materials.

## Experimental

### Materials

Polystyrene beads with a diameter of 400 nm suspended in an aqueous solution (1 wt %) were purchased from AGAR scientific Ltd. 20% ethanol and 80% de-ionized water were used for the dispersion of the beads. The substrates used were (100) oriented n-type silicon.

### Methods

#### Patterned substrate

To template the PS spheres, we used a sparse 2D array of posts fabricated by a scanning electron beam lithographic patterning of a 600 nm thick hydrogen silsesquioxane (HSQ) resist layer on a Si substrate. 20 patterns each with a size of 500 × 500 µm^2^ were fabricated on a Si substrate of 2 × 2 cm^2^. Si substrates were cleaned with acetone and then with a piranha solution for 24 h at 80 °C. After this step, the samples were left in a beaker containing ionized water for 12 h. The samples were dried immediately after use. HSQ is a radiation sensitive spin on glass which forms silica-like materials directly upon exposure to an electron beam. Development of these samples reveals the exposed posts, without requiring further etching or processing. We tried various diameters and pitches of the posts obtained by electron beam lithography and studied their influence on the self-organization of the beads. Diameters of these posts ranged from 195 nm to 420 nm with a height of 600 nm and the pitch varied between 5 to 20 times the diameters of the beads (Figure 1).

#### Self-organization of PS beads on patterned silicon substrate

PS beads were deposited on the silicon patterned substrates by a convective self-assembly technique. Figure 1 shows a schematic of the entire process. The ultrasonic cleaning of the patterned substrates was conducted in acetone for 5–10 min in order to remove the surface contamination. Thereafter, we applied 60 µL drops of PS beads (concentration 1 wt %). This concentration is chosen to ensure the formation of a dense monolayer structure after the evaporation of water.

#### Fabrication of silicon nanostructures produced by direct etching

Reactive ion etching (RIE) was implemented for the dry etching of the silicon surfaces by using the beads as a mask. The gases used for RIE were SF_6_ and O_2_. The regions covered by the beads are not etched by RIE. In this manner, we obtained a pattern on silicon. Beads with a diameter of 400 nm were used for patterning. The total etching time utilized was 4 min.

#### Characterization techniques

Optical microscopy, scanning electron microscopy (SEM) (HITACHI S-3400N, 30 kV), and associated Fourier transform analysis were employed to characterize the structure of the PS bead arrays on the patterned substrate.
